# Secondary Structure of Rat and Human Amylin across Force Fields

**DOI:** 10.1371/journal.pone.0134091

**Published:** 2015-07-29

**Authors:** Kyle Quynn Hoffmann, Michael McGovern, Chi-cheng Chiu, Juan J. de Pablo

**Affiliations:** 1 Institute for Molecular Engineering, University of Chicago, Chicago, Illinois, United States of America; 2 Department of Chemical Engineering, National Cheng Kung University, Tainan, Taiwan; 3 Argonne National Laboratory, Argonne, Illinois, United States of America; University of Leeds, UNITED KINGDOM

## Abstract

The aggregation of human amylin has been strongly implicated in the progression of Type II diabetes. This 37-residue peptide forms a variety of secondary structures, including random coils, α-helices, and β-hairpins. The balance between these structures depends on the chemical environment, making amylin an ideal candidate to examine inherent biases in force fields. Rat amylin differs from human amylin by only 6 residues; however, it does not form fibrils. Therefore it provides a useful complement to human amylin in studies of the key events along the aggregation pathway. In this work, the free energy of rat and human amylin was determined as a function of α-helix and β-hairpin content for the Gromos96 53a6, OPLS-AA/L, CHARMM22/CMAP, CHARMM22*, Amberff99sb*-ILDN, and Amberff03w force fields using advanced sampling techniques, specifically bias exchange metadynamics. This work represents a first systematic attempt to evaluate the conformations and the corresponding free energy of a large, clinically relevant disordered peptide in solution across force fields. The NMR chemical shifts of rIAPP were calculated for each of the force fields using their respective free energy maps, allowing us to quantitatively assess their predictions. We show that the predicted distribution of secondary structures is sensitive to the choice of force-field: Gromos53a6 is biased towards β-hairpins, while CHARMM22/CMAP predicts structures that are overly α-helical. OPLS-AA/L favors disordered structures. Amberff99sb*-ILDN, AmberFF03w and CHARMM22* provide the balance between secondary structures that is most consistent with available experimental data. In contrast to previous reports, our findings suggest that the equilibrium conformations of human and rat amylin are remarkably similar, but that subtle differences arise in transient alpha-helical and beta-strand containing structures that the human peptide can more readily adopt. We hypothesize that these transient states enable dynamic pathways that facilitate the formation of aggregates and, eventually, amyloid fibrils.

## Introduction

Molecular dynamics (MD) and Monte Carlo (MC) simulations [1] are widely used to study the structure of polypeptides. A number of advanced sampling techniques have been proposed to improve the efficiency of simulations. Examples include thermodynamic integration [2], umbrella sampling [3], parallel tempering [4], metadynamics [5,6], bias exchange metadynamics [7], and flux-tempered metadynamics [8].

Recently, these techniques have been applied to the study of protein folding for systems such as insulin [9], amylin [10–20], and amyloid fibrils [21]. The force field used to describe intermolecular interactions determines peptide structure. However, available force fields often display inherent biases in the secondary structures, as summarized in Table 1.

**Table 1:** Reported biases for selected force fields.

Name	Water Model	Year	α-helix Bias?	β Bias?	Other Notes
Amberff99 [22]	TIP3P [23]	2000	High [24,25]	Low [26]	
Amberff99sb [27]	TIP3P	2006	Low [28,29]	Low [30]	Models ILDN residues poorly [31]
Amberff99sb-ILDN [31]	TIP3P	2010		Low [29]	
Amberff03 [32]	TIP3P	2003	High [28,29,33,34]	Low [35]	
Amberff03* [29]	TIP3P	2009	High [28]	Low [35]	
Amberff03w [36]	TIP4P/2005 [37]	2010			Struggled with Ubiquitin [38]
CHARMM22 [39]	TIPS3P [23]	1998	High [28,34]	Low [26]	
CHARMM27 [40,41]	TIPS3P, TIP4P	2004	High [28,42,43]	Low [30]	
Gromos53a6 [44]	SPC [45]	2004	Low [33]		
OPLS-AA/L [46]	TIP4P	2001	Low [26,33,43]	High [43]^/^Low [30]	Favors Disordered states [30,43]

A value of low for α-helix bias indicates that the force field failed to form the experimentally expected α-helically structure or failed to remain stable when experiments suggested that an α-helix was stable. A high value for α-helix bias indicates that the force field formed an α-helix when experimental evidence suggests that an α-helix is disfavored. Similarly, β-Bias indicates biases related to forming parallel and anti-parallel β-sheets.

These biases can cause a secondary structure to misfold, despite experimental evidence for its stability [26,28,30,33,43,47,48]. Likewise, biases may prevent simulations from sampling experimentally relevant secondary structures with the correct probability.

Several recent studies have investigated force field biases, usually by comparison with experimental NMR data [35,38,49]. Such studies have been largely limited to folded proteins, and have not attempted to calculate the free energy of the molecule. Studies of disordered polypeptides have been scarce. Long molecular dynamics or parallel tempering simulations are often used to identify metastable states. However, both techniques suffer from significant drawbacks. Large free energy barriers can prevent sampling of metastable states and, for molecular dynamics simulations and umbrella sampling, the resulting free energy estimates can exhibit large errors. Molecular dynamics simulations only explore those regions of low free energy that are accessible on the time scale of a simulation, making difficult a comparison of free energies corresponding to secondary structures from different regions.

Bias exchange simulations facilitate quantitative comparison of free energy minima corresponding to different secondary structures [7]. Large free energy barriers can be overcome, thereby enabling sampling of favorable and unfavorable states. It is based on the metadynamics method [5], which, like most density-of-states based sampling techniques, relies on history-dependent biases to overcome barriers [50]. The choice of order parameter is essential; if slow degrees of freedom are left out, systematic biases in the free energy can arise [51]. Due to the complex dynamics of proteins, choosing a small set of order parameters to study secondary structure is difficult [15]. Each order parameter can be sampled in a different simulation box and, for metadynamics simulations, the time to explore the free energy surface increases exponentially with the number of order parameters that are incorporated in a calculation. Bias exchange simulations allow the use of many simultaneous order parameters. These help each other overcome barriers in free energy along order parameters that are orthogonal to the one being sampled in a distinct simulation box or window. For non-periodic order parameters, such as the α-helical content of a peptide, errors can accumulate near the boundaries [52]; such errors, however, can be avoided by modifying the shape of the history dependent potential according to methods that we have recently developed [53].

Amylin is an important test case for the evaluation of force fields, as it is known to adopt random coils, β-hairpins, and α-helices [54]. It has been strongly implicated in the development of Type II Diabetes Mellitus (T2DM) [55]. In over 90% of patients with type II diabetes [56–59], amyloid fibrils have been found in the β-cells of the Islets of Langerhans [60–63]. These fibrils are composed primarily of islet amyloid polypeptide [61,62] (known also as amylin or IAPP) in the form of primarily β-sheets [64–67]. The formation of these fibrils in the β-cells of the Islets of Langerhans cause membrane disruption [68,69] and cell death [70,71]. Furthermore, the presence of negatively charged membranes has been shown to accelerate the aggregation and membrane disruption processes [72,73]. The disruption of the membrane occurs prior to the formation of the mature fibrils [74]. Indeed, mature fibrils are of limited cytotoxicity compared to the early oligomers [69], suggesting that the early aggregates are the cytotoxic species.

Several sources suggest a causal relation between IAPP and Type II Diabetes. While humans, cats, and dogs readily develop the disease and form aggregates, species such as mice and rats do not typically form aggregates or display diabetes like symptoms [75]. However, when human IAPP (referred to as hIAPP) is transgenically expressed in mice and rats, fibrils are formed and symptoms typical of T2DM become manifest [76–78]. Furthermore, the addition of solutions of hIAPP oligomers and fibrils to healthy Pancreatic β-cells quickly results in cell death [69,71]. Finally, a mutation in the hIAPP gene has been linked to the early onset of T2DM and quicker rates of fibrillization [79,80].

Because of the rapid rate of aggregation, the detailed structures of hIAPP aggregates in their early stages are not fully understood. The structure of the monomer in solution has been studied by circular dichroism (CD) [54,64–66,81,82], molecular simulations [10–20], 2D-IR [83], and NMR [84]. It is believed to be mostly a random coil, with all residues above the cysteine bridge transiently adopting α-helical structures. Small sections adopt a β-hairpin structure. Residues 2–5 are held in a β-loop due to a cysteine bond between residues 2 and 7. CD [73], NMR [85,86] and EPR [87] studies show that hIAPP adopts an α-helical conformation as it binds to the phospholipid micelles as a monomer [88] in the head group region [89]. The spectra of this conformation decreases inversely with the increase of fluorescently labeled fibrillar structures and the formation of β-sheet structures [66]. However, the detailed structures of the oligomers and toxic intermediates are presently unknown.

Rat islet amyloid polypeptide (or rat amylin or rIAPP) provides a useful counterpart to human amylin. Despite differing by only six residues (Table 2), rat amylin does not form fibrils and, as mentioned earlier, rats do not display symptoms of T2DM [90]. Like hIAPP, rIAPP is predominantly random coil in solution [54,82,91], and transiently adopts an α-helical structure between residues 5–23. Upon binding to a negatively charged lipid micelle or bilayer, it forms an α-helix whose structure has been solved via NMR [92], Unlike human amylin, rIAPP does not form β-hairpins. This is thought to be due to the presence of three prolines, which disrupt the secondary structure in the C-terminus [93].

**Table 2:** Sequence and charges used for the simulation of rat and human amylin.

Residue Number	1 5 10 15 20 25 30 35
hIAPP Sequence	KCNTATCATQRLANFLV**H**SSNN**F**G**AI**L**SS**TNVGSNTY-NH_2_
Charge Used	++ +
rIAPP Sequence	KCNTATCATQRLANFLV**R**SSNN**L**G**PV**L**PP**TNVGSNTY-NH_2_
Charge Used	++ + +

Differences in sequence are bolded.

Extensive experimental information is available for rat and human amylin in solution, including IR spectra, NMR chemical shifts, and CD data. Results from simulations can be compared quantitatively to experiments, thereby providing a stringent test of available force fields. While amylin is predominantly random coil in solution, it also transiently samples α-helical states and small β-turns and hairpins. Therefore, in contrast to larger proteins where these three structural elements are stable, the free energy differences between distinct states are small and differ from the typical funnel view of the free energy of protein secondary structures. Bias exchange simulations allow us to sample a variety of random coils, α-helices, and β-hairpins. Furthermore, we can determine the free energy differences between clusters of these structures. In this work, we evaluate the ability of Amberff99sb*-ILDN [29,31,34,35], Amberff03w [36], CHARMM22/CMAP [40,41], CHARMM22* [34], Gromos96 53a6 [44], and OPLS-AA/L [46] to describe the structure of rat and human amylin in solution.

## Materials and Methods

### Simulation Parameters

Molecular dynamics simulations were run using the Gromacs 4.6.2 package [94]. The structures predicted from NMR by the Ramamaoorthy group for human [86] and rat [92] amylin on a micelle were used as the starting points for bias exchange simulations, which can quickly explore other, different configurations. In both human and rat amylin, the C-terminus is amidated. Only the +3 charge version of human amylin was simulated here. A disulfide bond is present between residues 2 and 7. The peptide was placed in the center of a 8.5 nm dodecahedral box and solvated with water. The force field and water model combinations considered for rat and human amylin are shown in Table 3. The water model used to parameterize each force field is chosen for each force field and water combination. In addition, three additional combinations were chosen to evaluate the effect nonstandard force field and water pairings can have: Amberff99sb*-ILDN with TIP4P, Amberff03w with TIP4P, and CHARMM22* with TIP4P. For hIAPP, Amberff03w with TIP4P was omitted because Amberff03w was optimized with TIP4P2005, not TIP4P. CHARMM22* with TIPS3P was also omitted since the TIP4P water model better predicted the NMR shifts for rIAPP.

**Table 3:** Force field and water combinations used to simulate human and rat amylin.

Name	Year of Publication	Water Models Used
Amberff99sb*-ILDN	2011	TIP3P, TIP4P
Amberff03w	2010	TIP4P, TIP4P2005
CHARMM22/CMAP	2004	TIPS3P
CHARMM22*	2011	TIPS3P, TIP4P
Gromos96 53a6	2004	SPC
OPLS-AA/L	2001	TIP4P

Four Cl^-^ were used to neutralize rIAPP, and three Cl^-^ were used to neutralize hIAPP. The energy was minimized first by using the steepest descent algorithm for 500 steps and a step size of 0.01 nm without constraints, and then for 10000 steps with all bonds constrained. The system was then equilibrated for 100 ps at 310 K at constant volume, and then for 10 ns at 310 K and a constant pressure of 1.0 bar. The velocity-rescaling temperature coupling [95] and Parrinello-Rahman [96,97] barostat were used for the equilibration simulations. The temperature was coupled separately for the solvent and protein. A 2 fs time step was used. Center of mass motion was removed every 10 ns. Electrostatic interactions were handled using a particle mesh Ewald method [98,99] (PME), with a r_coloumb_ value of 0.9 nm, an order of 4, 0.12 nm spacing, a tolerance of 1e-5, and 3D geometry. A long range dispersion correction was applied for both energy and pressure. Bonds involving hydrogen were constrained during the simulation using the LINCS [100] algorithm.

### Bias Exchange Simulations

Bias exchange metadynamics simulations [7] were run for 600 ns for all force fields except Amberff99sb*-ILDN/TIP3P for rat, which was run for 1000 ns in order to confirm convergence. A Fourier spacing of 0.33 was used for PME. The Nose-Hoover [101,102] algorithm was used for temperature control, with unit chain length. Protein and non-protein atoms were coupled separately. LINCS [100] was used to constrain the bonds involving hydrogen. The system was run in the canonical ensemble from the configuration obtained at the end of the NPT equilibration runs described above. The α_RMSD_ and anti-parallel β_RMSD_ [103] order parameters have been shown to allow efficient sampling of α-helical and β-sheet structures. A bias exchange simulation was run with four replicas: one unbiased box, a box biasing along the α_RMSD_ order parameter, a box biasing along β_RMSD_, and one box biasing on both α_RMSD_ and β_RMSD_. A Gaussian hill of height 0.1 kJ/mol and standard deviation 2.0 was deposited every 1 ps. The parameters used for the switching function in the RMSD collective variables were: r_0_ = 0.8, n = 8, and m = 12. The proteins were aligned as a single molecule at every step and periodic boundary conditions were then disabled for the subsequent RMSD calculations. Preliminary simulations showed that, without a boundary correction, significant artifacts accumulated near values of 0 RMSD. Here we emphasize that a standard invert method [52] is unable to correct for errors around 0 α_RMSD_ and 0 β_RMSD_, and such errors can be significant. To circumvent this problem, we used the boundary correction modification of McGovern et al. [53] to account for errors near the boundaries. A lower and upper boundary of 0 and 32 were used for both α_RMSD_ and β_RMSD_ for this method. After 200ns, a grid was implemented between collective variable values of -5 and 42 and with 2351 bins. A wall on the maximum distance between the backbone nitrogen of the third residue and the backbone carboxyl C on the 35^th^ residue was placed at 7.0 nm, with a kappa value of 100.0 kJ/mol. The backbone atoms of the protein used for RMSD were aligned. The boundary correction version [53] of PLUMED 1.3 [104] was used for simulations without a grid. This was further modified to support the use of a grid.

In order to calculate the free energy versus α_RMSD_ and β_RMSD_, the bias applied to the box sampling both α_RMSD_ and β_RMSD_ was averaged from the beginning of the bias exchange simulations using custom scripts. The free energy was calculated in a 160 by 160 grid of α_RMSD_ and β_RMSD_ points ranging from 0 to 32 in each dimension.

### Molecular Dynamics Simulations

For comparison with the bias exchange results, molecular dynamics simulations with the Amberff03w and Gromos96 53a6 force fields were run with rat amylin for 500 ns. The starting points were configurations found during the bias exchange simulation, including local free energy minima for the α-helix, β-hairpin, random coil, as well as other, unstable structures. Five starting configurations were used for Gromos96 53a6/SPC, and four for Amberff03w/TIP4P2005. The β_RMSD_ was then plotted as a function of α_RMSD_ and compared to the figures of the free energy vs. α_RMSD_ and β_RMSD_.

### NMR Calculations

Structures were taken every 10 ps from each of the bias exchange simulations for rat amylin. The NMR chemical shifts were calculated for the H_α_, CO, C_α_, H_N_, and C_β_ atoms using the SPARTA+ program [105]. The default random coil values from the SPARTA+ program were used. The average free energy of each bin in the free energy surface was calculated by averaging the NMR shifts of all structures belonging to that bin. The predicted secondary shift for each atom was then found by calculating the weighted average of the average secondary shift for each bin multiplied by the probability corresponding to its free energy. These results are compared to those from Williamson and Miranker [106] for amylin at 278 K, with the secondary shifts calculated by subtracting the random coil shifts from SPARTA+ from the shifts from in the Biological Magnetic Resonance Database under accession number 7311. The secondary shifts for H_N_ were calculated at 310 K using the experimental temperature coefficients reported by these authors; other temperature coefficients, however, are not available and we therefore can only compare our results to data at 278 K. In the case of H_N_, we were able to compare the errors between the predicted shifts and the experimental measurements at 310 K and 278 K, respectively. We found that the same force fields that performed well with the NMR values uncorrected for temperature perform well with the corrected values. Therefore, the experimental shifts at 278 K are used for C_α_, C_β_, H_α_, and N to compare with simulation predictions.

To ascertain the effect of temperature on our calculations, we conducted additional simulations with Amberff99sb*-ILDN with TIP3P, Amberff03w with TIP4P/2005, and CHARMM22* with TIP4P at T = 280, 290, 300, 310, and 320 K. The purpose of these additional calculations was to determine the average change in C_α_ shifts that one might expect in the range of temperatures encompassing the experimental measurements and at a physiological temperature. Five independent simulations were performed at each of these temperatures, starting from configurations belonging to local minima identified using bias exchange metadynamics. These simulations were conducted in the NPT ensemble with the same parameters used above in the equilibration procedure. After generating a 5 ns trajectory for each configuration, the average C_α_ shift over a 1 ns period was calculated for every residue at each of these temperatures. The mean differences between the average C_α_ shifts (averaged over all 37 residues and over five independent runs) at 310 K and at each of the other four temperatures T mentioned above are denoted by μ(T), and are shown in S1 Table.

A two-sided t-test was performed on the resulting data for all residues and all runs at a given temperature, with the null-hypothesis that the difference was 0. For all t-tests, we failed to find a statistically significant difference at a confidence level of 0.05. These results are shown in S1 Table. In summary, the results of our additional calculations indicate that there is no statistically significant difference between the shifts computed at 310 K and those observed at 280K. These results are consistent with the experimental observations of Soong et al., whose measurements of the circular dichroism signal, which depends on the secondary structure of rIAPP, are relatively unchanged between 273–313 K [107].

### Secondary Structure Calculations

The secondary structure of each frame was calculated using DSSP 2.04 [108,109]. For each element of secondary structure calculated by DSSP (for example, α-helix, β-sheet, β-bridge, etc.), the average fraction of structures corresponding to a given α_RMSD_ and β_RMSD_ bin was calculated. For each residue, the average fraction of structures containing a secondary structure element was calculated by weighting the average secondary structure fraction of that bin by the probability corresponding to the free energy of that bin. The average fraction of peptide in a secondary structure was found by averaging the average fraction of a residue in a secondary structure over all residues. These were then summed into two groups: helix (α-helix, 3_10_ Helix or π Helix) or strand (β-sheet or β-Bridge). This was performed because π and 3_10_ helices are often misclassified as α-helices, and because in DSSP β-Bridges correspond to the same hydrogen bonding pattern as β-Sheets, but are shorter in length.

## Results and Discussion

### Free Energy Maps

**Fig 1:**
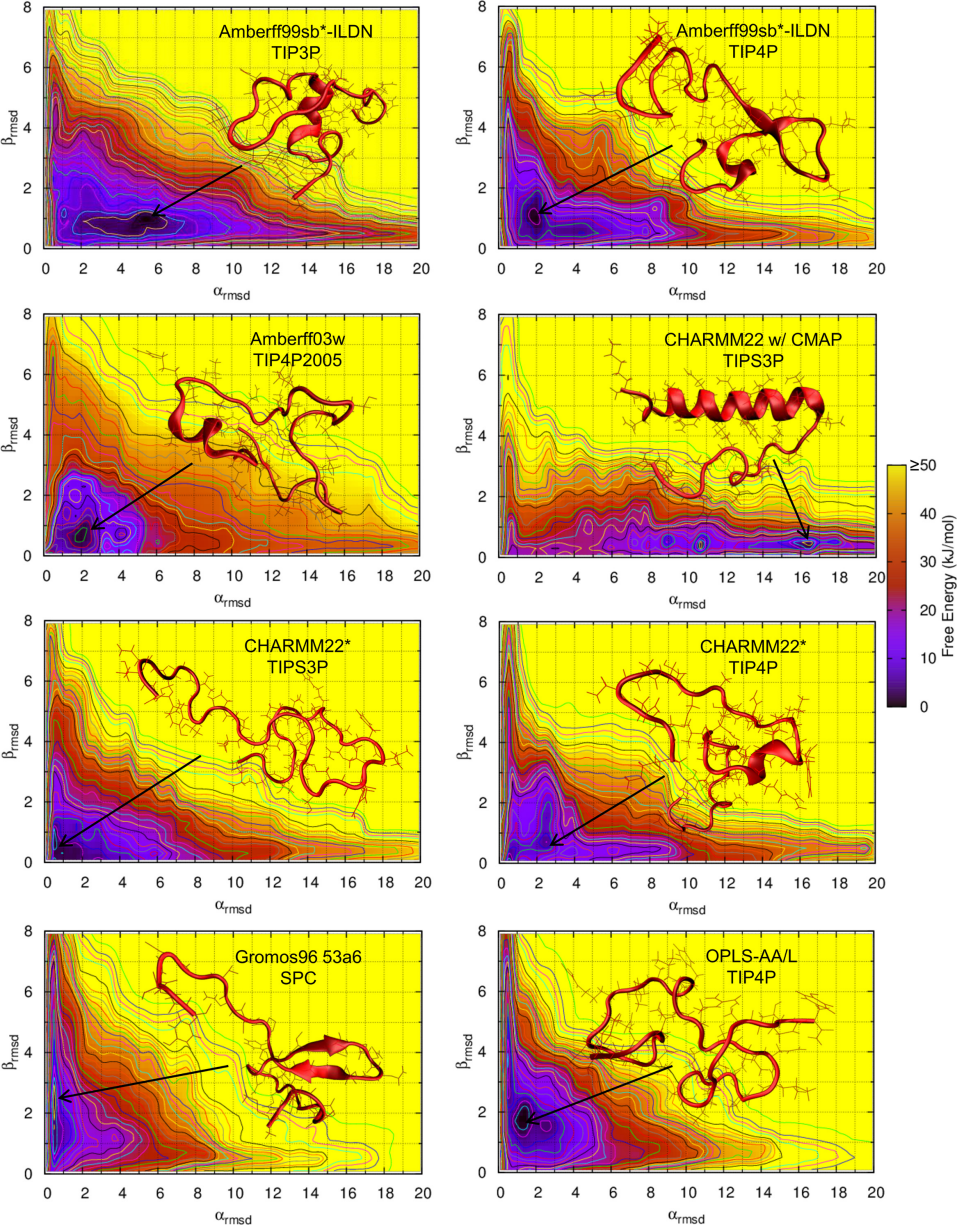
Free energy of rat amylin as a function of α_RMSD_ and β_RMSD_ for various force fields. The darker regions indicate regions of lower free energy.

The free energy of rIAPP as a function of α_RMSD_ and β_RMSD_ is shown in Fig 1 for various combinations of force fields and water models. Amberff03w with TIP4P is shown in S1 Fig. Note that α_RMSD_ and β_RMSD_ are proportional to the number of residues in the respective secondary structure; a larger value of α_RMSD_ indicates that a larger fraction of rIAPP is in an α-helical state. Once the α_RMSD_ increases beyond approximately 3 units, α-helices exist in a majority of structures. Likewise, β-sheets exist in most structures with a β_RMSD_ greater than approximately 2 units. The color of the plots provides the magnitude of the free energy in kJ/mol. Significant qualitative differences between many of the force fields considered here are immediately apparent. Most force fields favor a predominantly random coil, with the deepest local minima below 3 α_RMSD_ and 2 β_RMSD_. However, CHARMM22/CMAP heavily favors α-helical structures, with a deep minimum around 16 RMSD. In contrast, Gromos96 53a6 favors β-hairpin structures: its deepest minimum occurs around 0.5 α_RMSD_ and 2.5 β_RMSD_. The Amberff99sb*-ILDN force fields predict predominantly random coil structures. However, the α-helical and β-hairpin structures are not completely disfavored, and significant fractions of amylin exist in both states. Amberff03w with TIP4P2005 is almost completely disordered. There are no local minima in the α-helical or β-hairpin states. CHARMM22* with TIPS3P exhibits a deep minimum at 1 α_RMSD_ and 0.5 β_RMSD_, well within the random coil region. The OPLS-AA/L force field exhibits a qualitatively different free energy surface from those of the other force fields, with long α-helical and 2 β-hairpin structures being highly disfavored. It is, however, less α-helical and more heavily β-hairpin than Gromos96 53a6 with SPC. Importantly, these free energy differences are substantial and lead to qualitatively different conclusions regarding the pathways for amyloid formation.

**Fig 2:**
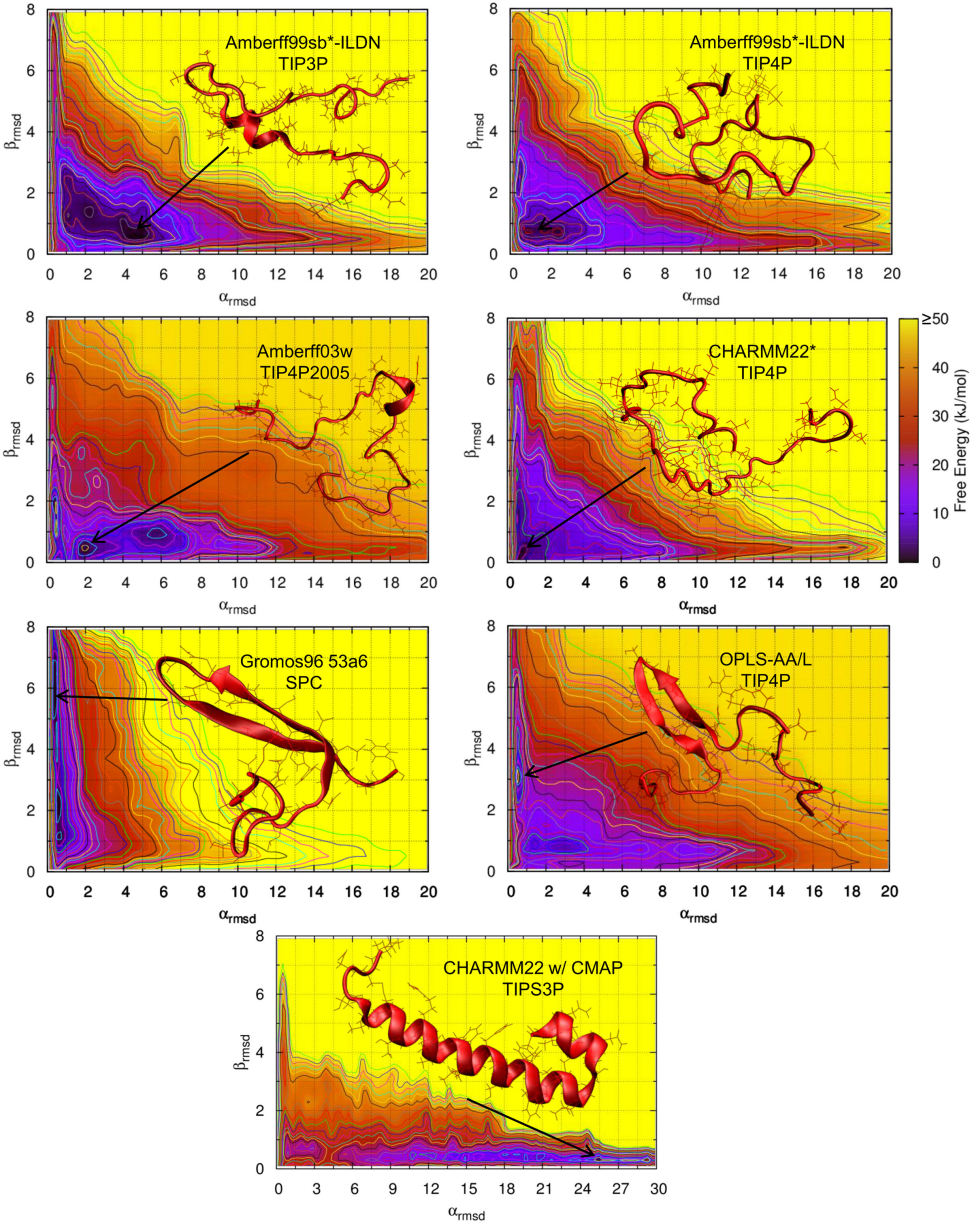
Free energy of human amylin as a function of α_RMSD_ and β_RMSD_ for various force fields. The darker regions indicate regions of lower free energy.

The free energy surfaces of human amylin are shown in Fig 2. The Gromos96 53a6 force field predicts much more β-sheet character than the other force fields—much greater than that predicted for rat amylin. The other force fields follow trends similar to those seen above for rIAPP: the Amber and CHARMM22* force fields predict mostly random coil structures with a small amount of α-helical and β-hairpin structures. OPLS-AA/L is also mostly random coil and predicts more β-sheet structures compared with rIAPP. The free energy of the β-sheet structures is also lower than that observed in all other force fields except Gromos96 53a6.

### Molecular Dynamics Simulations

**Fig 3:**
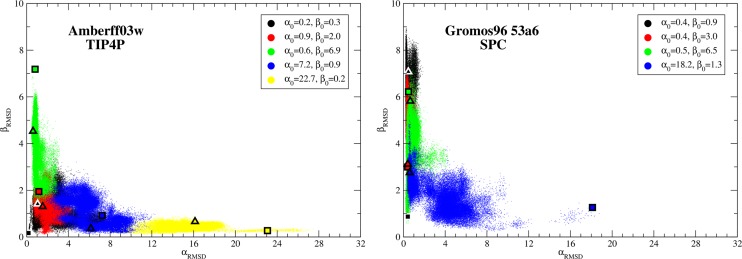
α_RMSD_ vs. β_RMSD_ every 10 ps of a molecular dynamics simulation of rIAPP starting at different configurations. The starting points are shown as squares while the ending points are shown as triangles. Each run was 500 ns long. The results for Amberff03w with TIP4P are shown on the left, while those for Gromos 96 53a6 are shown on the right.

Molecular dynamics simulations were run for 500ns starting from several selected structures for Amberff03w with TIP4P and Gromos96 53a6 with SPC. The α_RMSD_ and β_RMSD_ are shown in Fig 3 every 10 ps. The starting configurations are shown as squares and the ending configurations as triangles. As can be seen in the figure, the two force fields behave qualitatively different. The simulations for Amberff03w retain greater amounts of α-helical character than Gromos96 53a6. For example, structures starting in the high α-helical state retain large amounts of α-helical character throughout the 500 ns for Amberff03w. In Gromos96 53a6, the α-helices quickly unravel. In contrast, the large β-hairpins are unstable in Amberff03w while in Gromos96 53a6 a large β-hairpin forms from an initially low β_RMSD_ state.

These simulations are consistent with the free energy surfaces from bias exchange simulations discussed above. Amberff03w predicted a balance between α-helical and β-hairpin structures and indeed the molecular dynamics simulations drifted towards these free energy minima. In contrast, the Gromos96 53a6 simulations disfavor α-helical structures and prefer β-hairpin structures.

While the highly α-helical structure for Amberff03w remains in a high α_RMSD_ state predicted to be relatively unstable, this is most likely due to the slow conformational dynamics and not an inherent preference of the protein under this force field. This simulation provides evidence that caution is needed when analyzing MD simulations of proteins, even when half-microsecond simulations are considered. In this example, the underlying free energy landscape allows the peptide to remain trapped in relatively unfavorable conformations for at least 500 ns.

### Secondary Structures Predicted

**Fig 4:**
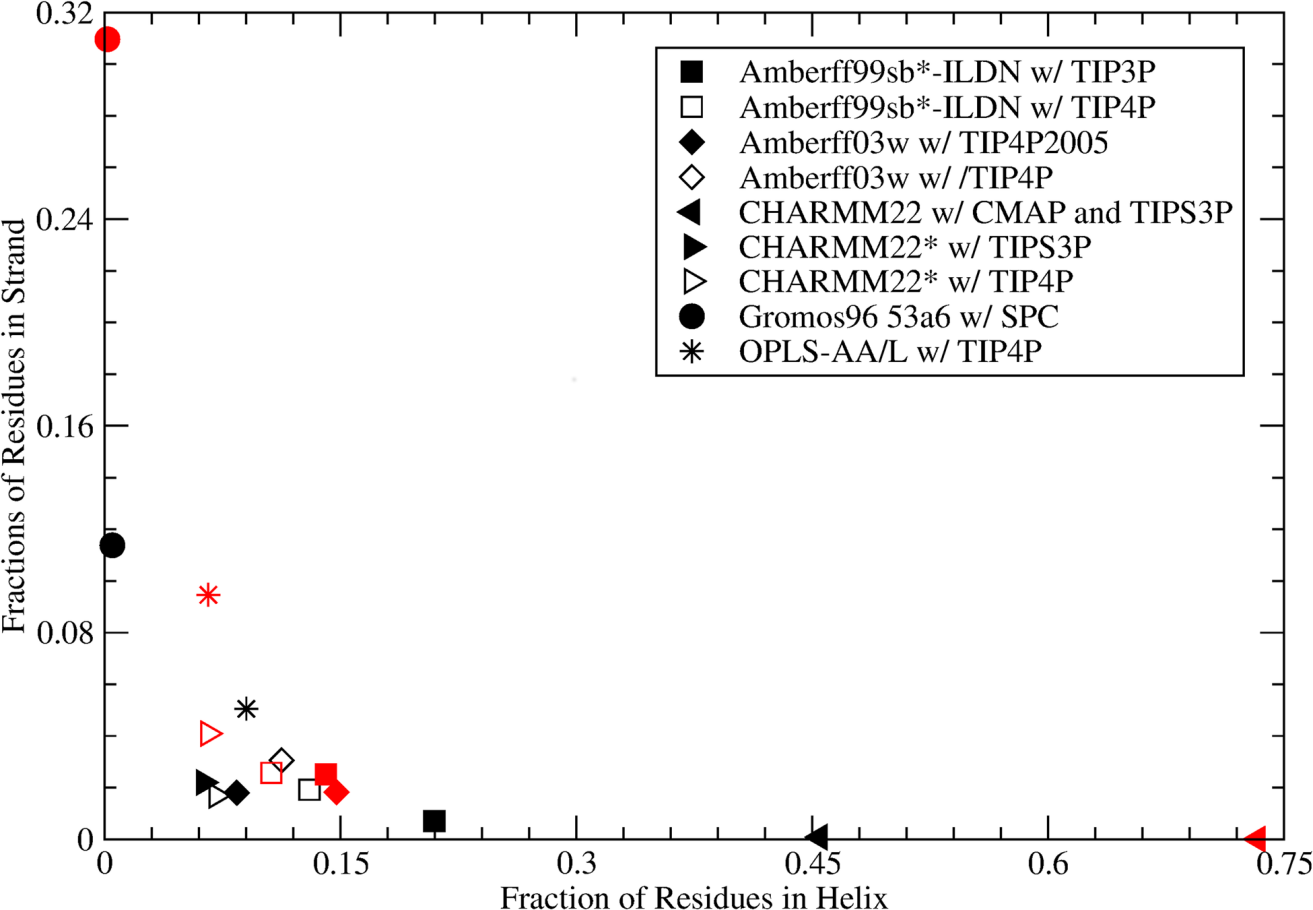
Fraction of structures with a helix (α-helix, 3_10_ Helix or π Helix) or a strand (β-sheet or β-Bridge) for rIAPP (black symbols) and hIAPP (red symbols) as predicted with various force fields. Force field and solvent model combinations where the force field was optimized with that solvent model are shown with a filled symbol; otherwise an unfilled symbol is shown.

The free energy surfaces were used to calculate the fraction of amino acids in a helix (α-helix, 3_10_ helix, or π-helix) or strand (β-sheet or β-bridge) as described above in the Methods section. The results at 310 K are shown in Fig 4 and span a range of helix and strand fractions. Depending on the force field, amylin can be found to adopt a helical structure with no β-sheet character, or a mostly random coil state with varying amounts of β-sheet structures. The force field which predicts the largest helical content for amylin is CHARMM22/CMAP with TIPS3P. Approximately 47% of amylin’s residues are predicted to be in an α-helix, 3_10_ helix, or π-helix structure. This is not surprising, as this force field has been previously noted to exhibit a strong α-helical bias [28,42,43]. The force field predicting the second highest helical content is Amberff99sb*-ILDN with TIP3P (21%). Gromos96 53a6 predicts less than 0.5% helical content and the highest strand content, namely 11%. The next highest prediction of β-sheet content is by the OPLS-AA/L force field with TIP4P, which yields 9% helical content and 5% strand content.

The difference in structural propensities between human and rat amylin predicted depends on the force field chosen. For Gromos96 53a6, for example, the fraction of residues in a β-sheet or β-bridge conformation is 0.31 for human versus 0.11 for rat. OPLS-AA/L predicts a change of this fraction of residues in a strand conformation from 0.095 in hIAPP to 0.051 in rIAPP, while CHARMM22* with TIP4P predicts the fraction changing from 0.041 to 0.017 for human and rat respectively. In contrast, Amberff03w’s strand content is 0.018 for both hIAPP and rIAPP, but the helical fraction is 0.15 in hIAPP compared to 0.084 in rIAPP. The amount of helical content is almost identical to that observed by Miller et al. [12] in replica exchange simulations with the same force field and water model (0.15 for hIAPP, and 0.086 for rIAPP). However, Miller et al. predicts more strand content in rIAPP and hIAPP (0.053 for rIAPP and 0.038 for hIAPP). The small differences in secondary structure content may be the result of differences in temperature, the amidation state of the C-terminus, or salt concentration. For most other force fields, the amount of helical character in human amylin is smaller than the amount of strand character, and the difference in the predicted number of residues in an α-helical or β-hairpin character between hIAPP and rIAPP depends on force field.

The predicted strand and helix fractions of rat amylin for different water models are also shown in Fig 4. Three non-standard water models were investigated: Amberff99sb*-ILDN w/ TIP3P and TIP4P, Amberff03w with TIP4P and TIP4P2005, and CHARMM22* with TIPS3P and TIP4P. The corresponding free energies relative to the coil state are shown in Table 4. For Amberff99sb*-ILDN, the use of TIP4P instead of the recommended TIP3P results in an increase in the free energy of the helix state for the average residue of 0.6 kT relative to the coil structure. The free energy of the strand fraction decreased by 0.9 kT. These differences correspond to a change in fraction of residues in a helix and strand of -0.08 and 0.01 respectively. For Amberff03w, the free energy of the helix states and strand states decreased by 0.4 and 0.6 kT, corresponding to increases in fraction of residues of 0.03 and 0.01, respectively. In CHARMM22*, the use of TIP4P instead of TIP3P changed the free energies by -0.1 and 0.2 kT, altering the fractions of helix and strand by +0.01 and -0.005, respectively. Amberff99sb*-ILDN exhibits the most dramatic free energy change, where the fraction of helix decreases by almost half, bringing its predictions for helix into rough agreement with those of Ambeff03w, CHARMM22*, and OPLS-AA/L. Its strand content also increases to 0.02, in agreement with these force fields. In contrast CHARMM22*’s secondary structure composition remains roughly unaltered. This suggests that different water models may be used with relatively little ill effect on CHARMM22*, as already suggested by Bjelkmar et al. [41]. Amberff03w also exhibits a slight decrease in strand and helix content, making it very similar to CHARMM22*’s predictions.

**Table 4:** Free energy of secondary structure appearing in a residue in rIAPP relative to the coil secondary structure in units of kT at 310 K.

Force Field	Water Model	Helix	Strand	Turn	Bend
Amberff99sb*-ILDN	TIP3P	0.52	3.91	0.47	0.53
Amberff99sb*-ILDN	TIP4P	1.12	3.03	0.51	0.63
Amberff03w	TIP4P2005	1.72	3.26	1.24	0.47
Amberff03w	TIP4P	1.36	2.67	1.00	0.54
CHARMM22/CMAP	TIPS3P	-0.47	5.84	1.08	0.56
CHARMM22*	TIPS3P	2.12	3.17	1.39	0.70
CHARMM22*	TIP4P	2.00	3.43	1.38	0.71
Gromos53a6	SPC	4.66	1.53	1.91	0.64
OPLS-AA/L	TIP4P	1.57	2.15	1.09	0.42

**Fig 5:**
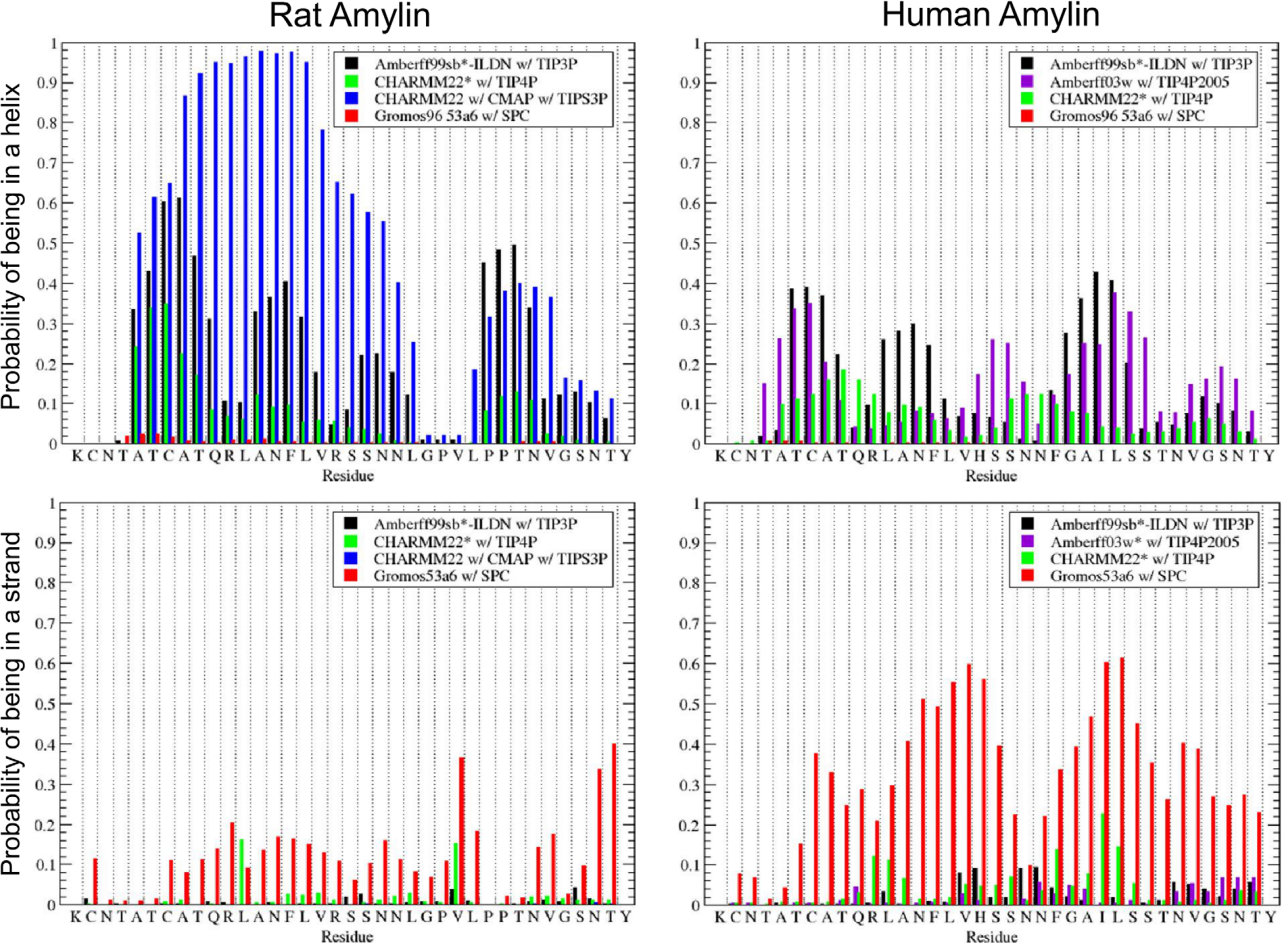
Predicted fraction of time each residue is in a helix (α-helix, 3_10_ Helix or π Helix) or strand (β-sheet or β-Bridge). The predicted fractions for rat are shown on the left for the Amberff99sb*-ILDN with TIP3P, CHARMM22* with TIP4P, CHARMM22/CMAP with TIPS3P, and Gromos96 53a6 with SPC force fields. On the right, the predicted fractions of human amylin are shown for Amberff99sb*-ILDN with TIP3P, Amberff03w with TIP4P, CHARMM22* with TIP4P, and Gromos96 53a6 with SPC.

These overall helix and strand propensities are broken down by residue in Fig 5. CHARMM22/CMAP exhibits the most helical behavior. This is concentrated between residues 5–23 as observed in experiments and secondary structure prediction algorithms. For this force field, these residues are usually in an α-helix. This α-helix is broken by glycine at residue 24. Another helix occasionally forms in the tail between residues 27 and 36. This is predicted to be present roughly 30% of the time by CHARMM22/CMAP. Amberff99sb*-ILDN is the next most helical force field. It follows the same broad trends with intermittent helices between residues 5–23 and 27–36. These are followed by Amberff03w with TIP4P2005 and CHARMM22* with TIPS3P. CHARMM22* predicts a helix more frequently between residues 27–36 than between 7–23.

Gromos96 53a6 with SPC has the largest strand propensities by residue. These are relatively evenly distributed between residues 7–36, with a break at the double prolines at residues 28 and 29. In general, the strand is predicted to be much less frequent than the helix. Amberff99sb*-ILDN with TIP3P has very little strand propensity throughout. Amberff03w and CHARMM22* have roughly equal strand propensities.

### Convergence

**Fig 6:**
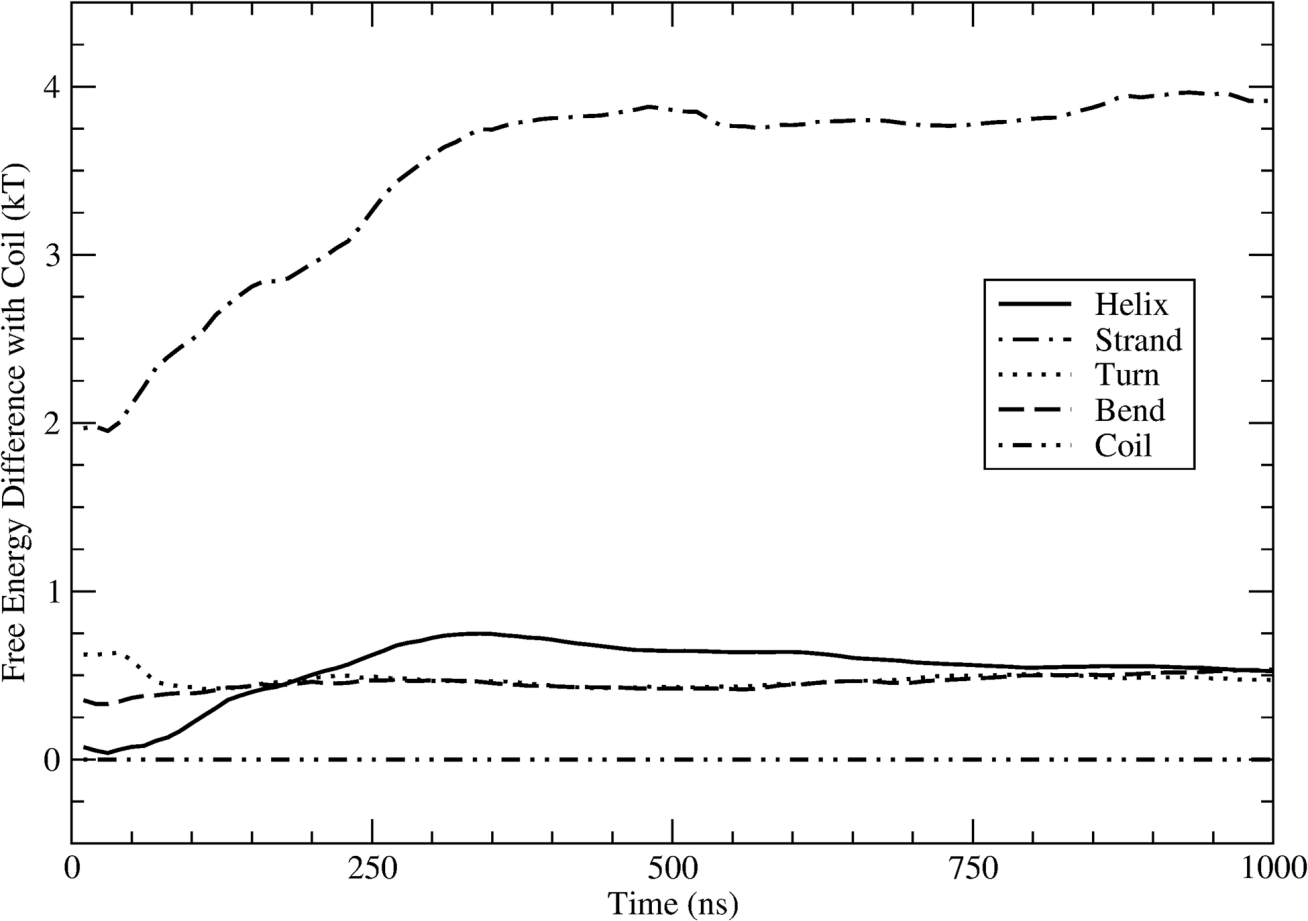
Free energy difference with coil for fraction of residues in a secondary structure predicted for rIAPP vs. time for Amberff99sb*-ILDN with TIP3P. The secondary structure of each residue was determined using DSSP.

Fig 6 shows the free energy of rIAPP in a helix, strand, turn, and bend (relative to the coil) versus time for Amberff99sb*-ILDN with TIP3P. The free energy of the bend and turn states converges within approximately 100 ns, reaching a value that is 0.5 kT greater than the coil state. The helix and strand states take longer to converge. The difference in free energy between the helix and coil states starts small but grows to a maximum of 0.75 kT before slowly decreasing. At the end of the simulation the difference is 0.53 kT. The free energy difference between strand and coil increases rapidly until about 350 ns when it reaches a value of 3.8 kT. It then fluctuates around this value for the rest of the simulation, finishing at 3.9 kT. While the fraction of helix and possibly strand may not have completely converged even after 1000 ns, as evidenced by the small increase in fraction of helix, the differences are relatively unchanged after 350 ns. When these free energy differences are converted to fractions of the structure that are in a helix or strand versus time, they become even smaller and remain within 0.05 of the value at 100 ns. These fractions are the important weights when determining properties of the molecule, such as NMR shifts, hydrogen bonding, and chemical reaction rates. Small changes of less than 0.05 fraction of these structures will not greatly influence the results. Furthermore, these changes are small compared to the large differences between the force fields. The convergence plots for the other force fields are shown in S2 and S3 Figs for rIAPP and hIAPP respectively.

### NMR Secondary Shifts

**Fig 7:**
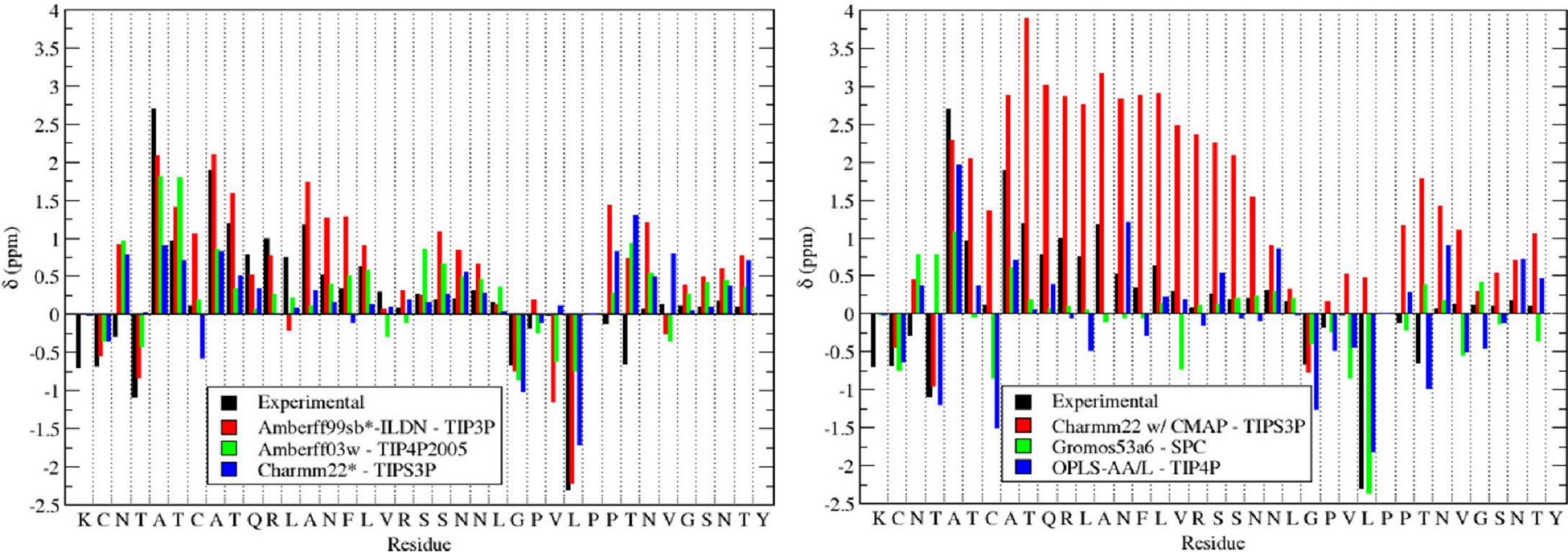
Predicted C_α_ secondary chemical shifts for Amberff99sb*-ILDN with TIP3P, Amberff03w with TIP4P2005, CHARMM22* with TIPS3P, CHARMM22/CMAP with TIPS3P, Gromos96 53a6 with SPC, and OPLS-AA/L with TIP4P. The predictions were made using SPARTA+.

The NMR secondary shifts were computed for amylin and compared with the experimental results from Williamson and Miranker [106]. These were calculated for the C_α_, C_β_, H_α_, H_N_, and N atoms. The random coil shifts from SPARTA+ [110] were used for these calculations. These random coil shifts were subtracted from the chemical shifts obtained by Williamson and Miranker in order to compare secondary shifts calculated using the same random coil shifts. The C_α_ secondary shifts provide a measure of the secondary structure propensity of the residue; a value greater than 0 suggests a propensity towards helical structures; a value of 0 indicates a random coil structure, and values less than 0 correspond to β-sheet structures. The C_α_ secondary shifts are shown in Fig 7 for several force field and water combinations. Amberff99sb*-ILDN with TIP3P qualitatively reproduces the major trends in the chemical shift pattern. Residues 5–25 are up-shifted, corresponding to the increased α-helical propensity. It also captures the down field secondary shifts around the glycine and first proline. The agreement with the experimental results is the poorest at the C-terminus. The values predicted are higher than the experimental chemical shifts, corresponding to a slight propensity in the simulations to form a small α-helix at the C-terminus. Most experimental evidence suggests that this region is predominantly unstructured. Because random coil regions involve a large number of low energy states their sampling is challenging and prone to statistical uncertainties, which may explain the differences between the predicted and experimental results. Fig 7 shows a similar result for Amberff03w with TIP4P2005 and CHARMM22* with TIPS3P: the discrepancy between the predicted and experimental NMR results is greatest in the disordered C-terminus of rIAPP.

The second half of the figure shows the shifts for CHARMM22/CMAP with TIPS3P, Gromos53a6 with SPC, and OPLS-AA/L with TIP4P. CHARMM22/CMAP with TIPS3P is drastically upshifted of the experimental results as a result of its high helix propensity. In contrast, Gromos96 53a6, which does not predict significant amounts of α-helical character, consistently underestimates the C_α_ coefficients at the N-terminus. OPLS-AA/L with TIP4P is the most downfield shifted and fails to reproduce the NMR shifts at both the N-terminus and C-terminus.

The average error for the predicted NMR shifts is shown in Table 5 in units of ppm / residue. They were calculated using the following formula:

$$error=\frac{1}{n}\overset{n}{\underset{i=1}{\sum }}\left|\delta_{i,pred}-\delta_{i,ex}\right|$$

where n is the number of residues, and δ_i,pred_ and δ_i,ex_ are the predicted and experimental shifts for the i^th^ residue respectively. The best performing force fields are Ambeff99sb*-ILDN, Amberff03w, and CHARMM22*. For Amberff99sb*-ILDN, the secondary shifts with TIP4P are better than with the recommended water model TIP3P. This may correspond to the lower overall α-helical content of this model. With Amberff03w, the C secondary shifts are much lower with TIP4P2005 than with TIP4P, suggesting that the distinction between the two water models may be important. CHARMM22* with TIP4P produces the lowest errors for the C and H chemical shifts, even compared to the recommended water model TIPS3P. This agrees with the suggestion by the authors of the Gromacs implementation of the CHARMM22/CMAP force field that TIP4P may be a preferable alternative to TIPS3P [41]. Surprisingly, Gromos96 53a6 has the smallest error for the backbone nitrogen atoms, while also showing large errors for the carbon and hydrogen secondary shifts.

**Table 5:** Average error in the NMR secondary shifts (ppm/residue) predicted for rIAPP for different force field and water models at 310 K.

Force Field	Water Model	C_α_	C_β_	H_α_	H_N_	N
Amberff99sb*-ILDN	TIP3P	0.52	0.42	0.10	0.12	2.64
Amberff99sb*-ILDN	TIP4P	0.47	0.39	0.10	0.09	2.34
Amberff03w	TIP4P	0.62	0.44	0.11	0.12	1.48
Amberff03w	TIP4P2005	0.51	0.38	0.11	0.14	1.44
CHARMM22/CMAP	TIPS3P	1.24	0.49	**0.09**	0.10	2.43
CHARMM22*	TIPS3P	0.51	0.40	0.12	0.13	1.20
CHARMM22*	TIP4P	**0.38**	**0.36**	0.10	**0.09**	1.38
Gromos53a6	SPC	0.55	0.50	0.16	0.24	**0.99**
OPLS-AA/L	TIP4P	0.54	0.45	0.14	0.10	2.09

The smallest percent error is in bold for each secondary shift.

### Comparison of Human and Rat Amylin

As summarized in Fig 4, the fraction of amylin predicted to adopt a helical or strand conformation varies appreciably across force fields. Most force fields predict that both human and rat adopt a predominantly random coil structure in solution, consistent with experiments. CHARMM22* with TIP4P, the force field that most accurately predicted the NMR shifts, predicts that the strand content is twice as large in human versus rat amylin, though it remains below 5%. For Amberff03w with TIP4P2005, rat and human amylin exhibit approximately equal fractions of strand character, consistent with the results of Miller et al. [12]. All other force fields predict modest increases in the amount of strand character. However, except for Gromos96 53a6, the strand content remains a small fraction of the overall secondary structure of the peptide.

For several key residues, however, the fraction of time spent in a helical or strand state differs between rat and human amylin, as seen in Fig 5. In rat amylin, little helical content is predicted between residues 24–26. Despite varying estimates of the overall amount of α-helical character between force fields, this decrease in helical propensity is consistently observed across all force fields examined here, even for CHARMM22/CMAP with TIP3SP. This is consistent with the tendency of proline to cap α-helices [111], and with NMR data indicating that the N-terminus α-helix ends with Leucine-23 [92]. In contrast, the helical propensity does not decrease sharply at these residues in human amylin. The aggregation of α-helices has been proposed to be an important intermediate in the formation of oligomers [112]. The ability of human amylin to form α-helices throughout the peptide could increase its propensity to form oligomers composed of helices. Larger helices could also be involved in oligomeric states along the pathway to the formation of amyloid fibrils in hIAPP; rIAPP may not be able to form these intermediate states composed of helices past residue 23.

Similarly, as shown in Fig 5, the double proline at residues 28 and 29 disrupts the β-sheet and β-bridge character for rat amylin, consistent with studies showing that prolines disrupt β-sheets [111]. While the strand content of rIAPP is generally low, no β-sheets are ever observed involving these prolines. However, for hIAPP, strand character is observed across these residues. Gromos96 53a6, with the largest prediction of β-sheet character, provides the most clear observation of this trend. In Amberff99sb*-ILDN with TIP3P or CHARMM22* with TIP4P, hIAPP occasionally forms β-sheets involving these residues, in contrast to rIAPP where no strand is ever observed involving residues 28 and 29. The ability to form β-sheets in this region agrees with experimental [75] and computational [20] evidence indicating that fibril formation is greatly inhibited when the peptide contains a proline at residue 25, 28, and/or 29.

**Fig 8:**
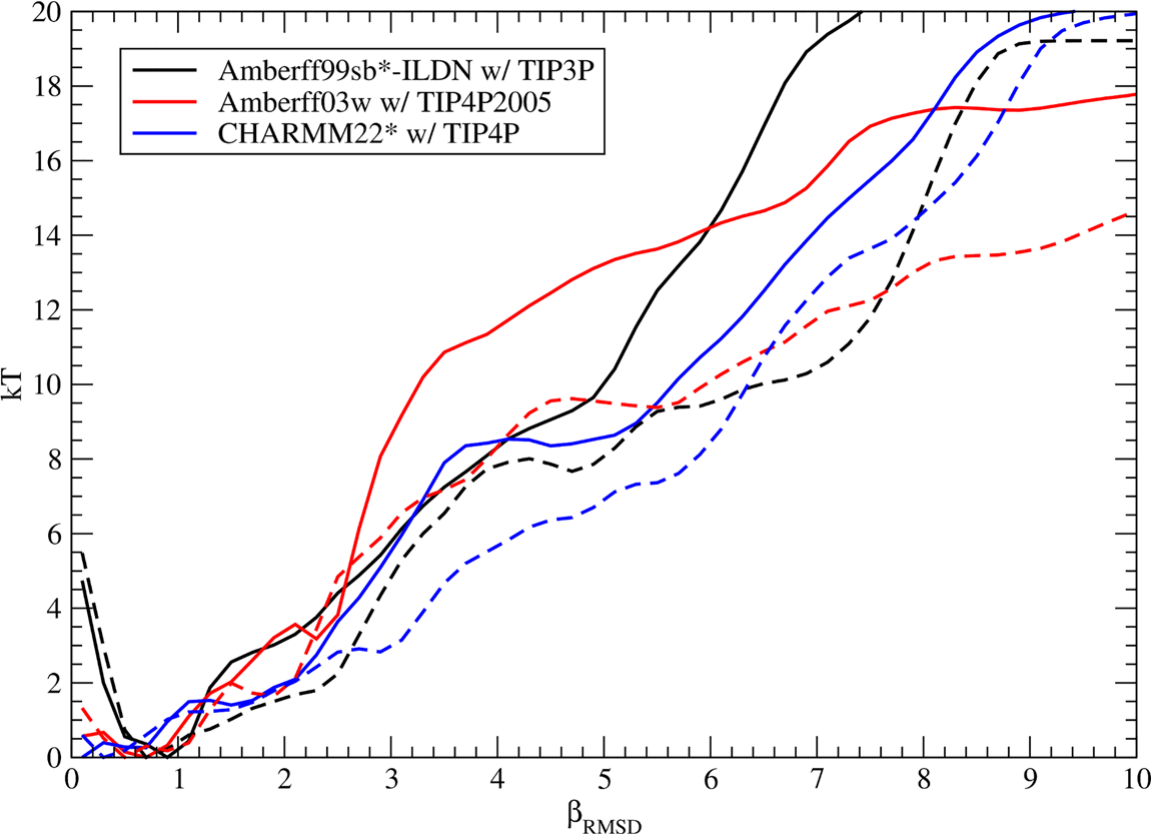
Helmholtz free energy in kT versus β_RMSD_ for rat and human amylin. The β_RMSD_ is correlated with the number of residues in a β-hairpin. The results for rIAPP are shown using solid lines, while the results for hIAPP are given in dashed lines. The Helmholtz free energy is shown for Amberff99sb*-ILDN with TIP3P (black), Amberff03w with TIP4P2005 (red), and CHARMM22* with TIP4P (blue).

The absence of this decrease in strand propensity is reflected in the formation of larger β-hairpins in hIAPP compared to rIAPP. Fig 8 shows the free energy versus β_RMSD_ of rIAPP and hIAPP for Amberff99sb*-ILDN with TIP3P, Amberff03w with TIP4P2005, and CHARMM22* with TIP4P. When the free energy along the α_RMSD_ coordinate is integrated out, a noticeable trend appears. In all three cases, when the β_RMSD_ is large, as in the case of a large hairpin, the free energy of hIAPP is less than that of rIAPP. We hypothesize that the ability of human amylin to form α-helices and β-hairpins across residues 24–29 allows the formation of a much more diverse set of potential transition structures, which could be responsible for the propensity of hIAPP to form fibrils. Indeed, for both the 2-fold [113] and 3-fold [93] models for amylin, large β-sheets are present. Furthermore, residues 28 and 29, which had no observed strand content in rIAPP but occasionally formed β-sheets for hIAPP, are predicted to be part of the β-strands experimentally observed in hIAPP. These residues are in the β-sheet region of conformations identified as intermediates in the formation of amyloid fibrils [114]. The formation of a β-hairpin involving these atoms has also been identified as part of a transition from the α-helical to β-hairpin conformations of hIAPP [115].

## Conclusions

The free energy of amylin was calculated as a function of α_RMSD_ and β_RMSD_ and used to determine the fraction of rat and human amylin in a helix or strand for various force field and water combinations. These results show that the choice of force field greatly influences the fraction of helix and strand, even for recent force fields such as Amberff99sb*-ILDN and CHARMM22*. Older force fields such as CHARMM22/CMAP and Gromos96 53a6 have strong biases towards α-helices and β-hairpins respectively. More elaborate calculations concerning the formation of dimers and oligomers are likely to be affected as well. Depending on the biases of the force field being used, the favorability of certain fibril precursors is likely influenced by the force field choice. CHARMM22* with TIP4P predicted the experimental rIAPP NMR secondary shifts most accurately. There was no general trend in the change of overall fraction of helix and strand between rat and human amylin across the different force fields. While Gromos96 53a6 predicts a large increase in β-hairpin character, CHARMM22* with TIP4P predicts only a small increase. Amberff03w with TIP4P2005, however, predicts a slight increase in helical character. Despite these differences, all force fields agree that the prolines in rIAPP cap helices in the C-terminus and disrupt the β-hairpins.

Taken together, the results of simulations of amylin across force fields present a picture in which the human and rat versions of the peptide are intrinsically disordered in solution, with only subtle structural differences between them. The human peptide, however, can more readily form transient α helices and β strands, which in the rat version are disrupted by the prolines at residues 25, 28 and 29. We hypothesize that these transient states confer to the human peptide a greater propensity to adopt β sheet structures that are stabilized by the presence of other peptides in aggregates or fibrils.

More generally, the study presented here represents a first systematic attempt to examine the structure and free energy of large disordered peptides across multiple force fields. Our results demonstrate that, for amylin, the preference towards α-helices, β-hairpins, or random coils is sensitive to the force field chosen; because of its intrinsically disordered nature, accurate force fields are essential in order to make predictions of secondary structure. In light of these findings, past simulations involving amylin (including our own) should be re-examined in the context of the force field chosen, especially when the resulting predictions align with given force field’s biases. Furthermore, the same issues could arise for other amyloid forming disordered peptides. A second note of caution is that the secondary structure of amylin was observed to change slowly. For example, in Gromos96 53a6, helices predicted to be unfavorable, remained relatively stable even for 500 ns. In light of this and other evidence that protein folding can take on the order of microseconds [116], the stability of a structure over a few hundred nanoseconds does not necessarily indicate that it corresponds to equilibrium.

## Supporting Information

S1 Table Average difference in ppm predicted for each residue between 310 and 280 K. The corresponding p-values are shown. Using the standard deviation of the means, and a power of 0.95, the predicted difference in ppm between the two temperatures that could be resolved is shown as Δ.
(DOCX)

S1 Fig Free energy of rat amylin as a function of α_RMSD_ and β_RMSD_ for Amberff03w with TIP4P. The darker regions indicate regions of lower free energy.
(TIFF)

S2 Fig Free energy difference with coil for fraction of residues in a secondary structure predicted for rIAPP vs. time for various force fields. The secondary structure of each residue was determined using DSSP.
(TIFF)

S3 Fig Free energy difference with coil for fraction of residues in a secondary structure predicted for hIAPP vs. time for various force fields. The secondary structure of each residue was determined using DSSP.
(TIFF)
