# Supplementary material for: Secondary Structure of Rat and Human Amylin across Force Fields
Source: PLoS One. 2015 Jul 29;10(7):e0134091. doi: 10.1371/journal.pone.0134091 (PMC4519342; doi:10.1371/journal.pone.0134091)
Supplement: S1 Table — The corresponding p-values are shown. Using the standard deviation of the means, and a power of 0.95, the predicted difference in ppm between the two temperatures that could be resolved is shown as Δ. (DOCX) [file pone.0134091.s001.docx]

S1 Table. Average difference in ppm predicted for each residue between 310 and 280 K.

| Force Field | Water Model | Temperatures | μ (ppm) | p-value | Δ (ppm) |
| --- | --- | --- | --- | --- | --- |
| Amberff99sb*-ILDN | TIP3P | 320-310 K | 0.12 | 0.07 | 0.32 |
|  |  | 310-300 K | -0.07 | 0.37 | 0.38 |
|  |  | 310-290 K | -0.08 | 0.26 | 0.31 |
|  |  | 310-280 K | -0.10 | 0.12 | 0.38 |
| Amberff03w | TIP4P2005 | 320-310 K | 0.03 | 0.57 | 0.31 |
|  |  | 310-300 K | 0.01 | 0.87 | 0.26 |
|  |  | 310-290 K | -0.09 | 0.11 | 0.30 |
|  |  | 310-280 K | -0.05 | 0.39 | 0.26 |
| CHARMM22* | TIP4P | 320-310 K | -0.04 | 0.34 | 0.23 |
|  |  | 310-300 K | 0.03 | 0.61 | 0.27 |
|  |  | 310-290 K | 0.06 | 0.24 | 0.24 |
|  |  | 310-280 K | 0.05 | 0.33 | 0.27 |

The corresponding p-values are shown. Using the standard deviation of the means, and a power of 0.95, the predicted difference in ppm between the two temperatures that could be resolved is shown as Δ.
