# Supplementary figures and images for: Secondary Structure of Rat and Human Amylin across Force Fields
Source: PLoS One. 2015 Jul 29;10(7):e0134091. doi: 10.1371/journal.pone.0134091 (PMC4519342; doi:10.1371/journal.pone.0134091)

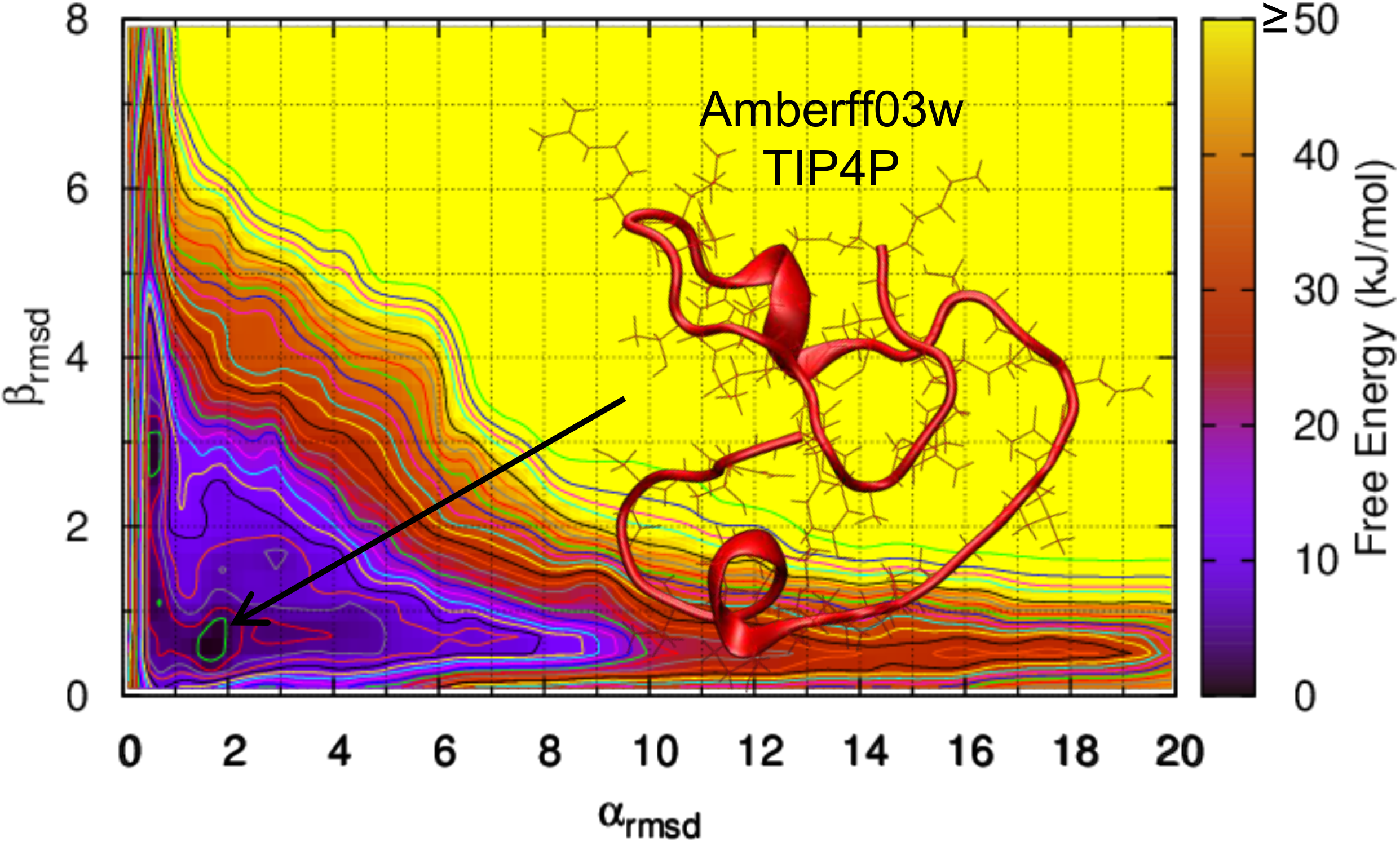

Supplement: S1 Fig — The darker regions indicate regions of lower free energy. (TIFF) [file pone.0134091.s002.tiff]

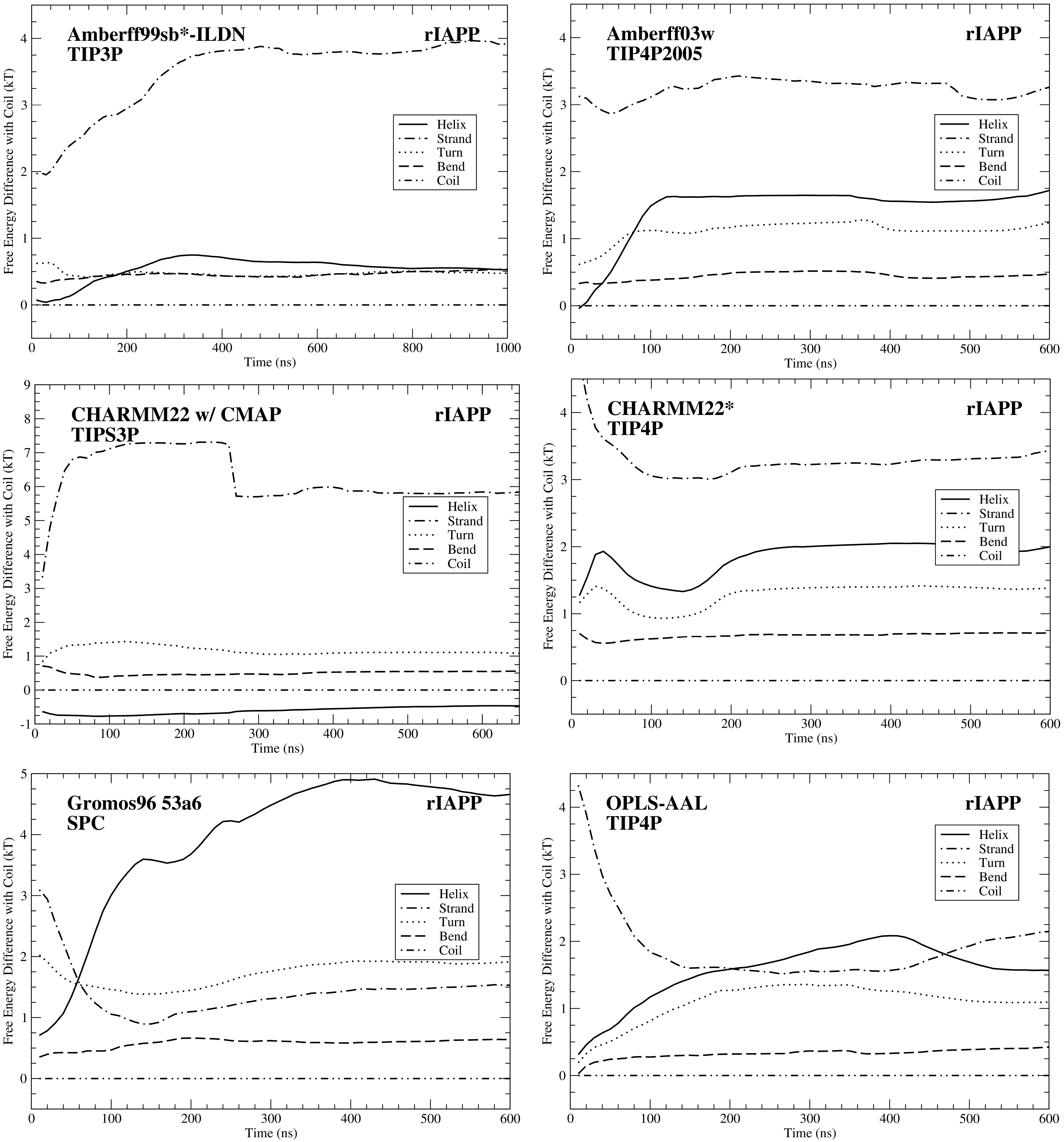

Supplement: S2 Fig — The secondary structure of each residue was determined using DSSP. (TIFF) [file pone.0134091.s003.tiff]

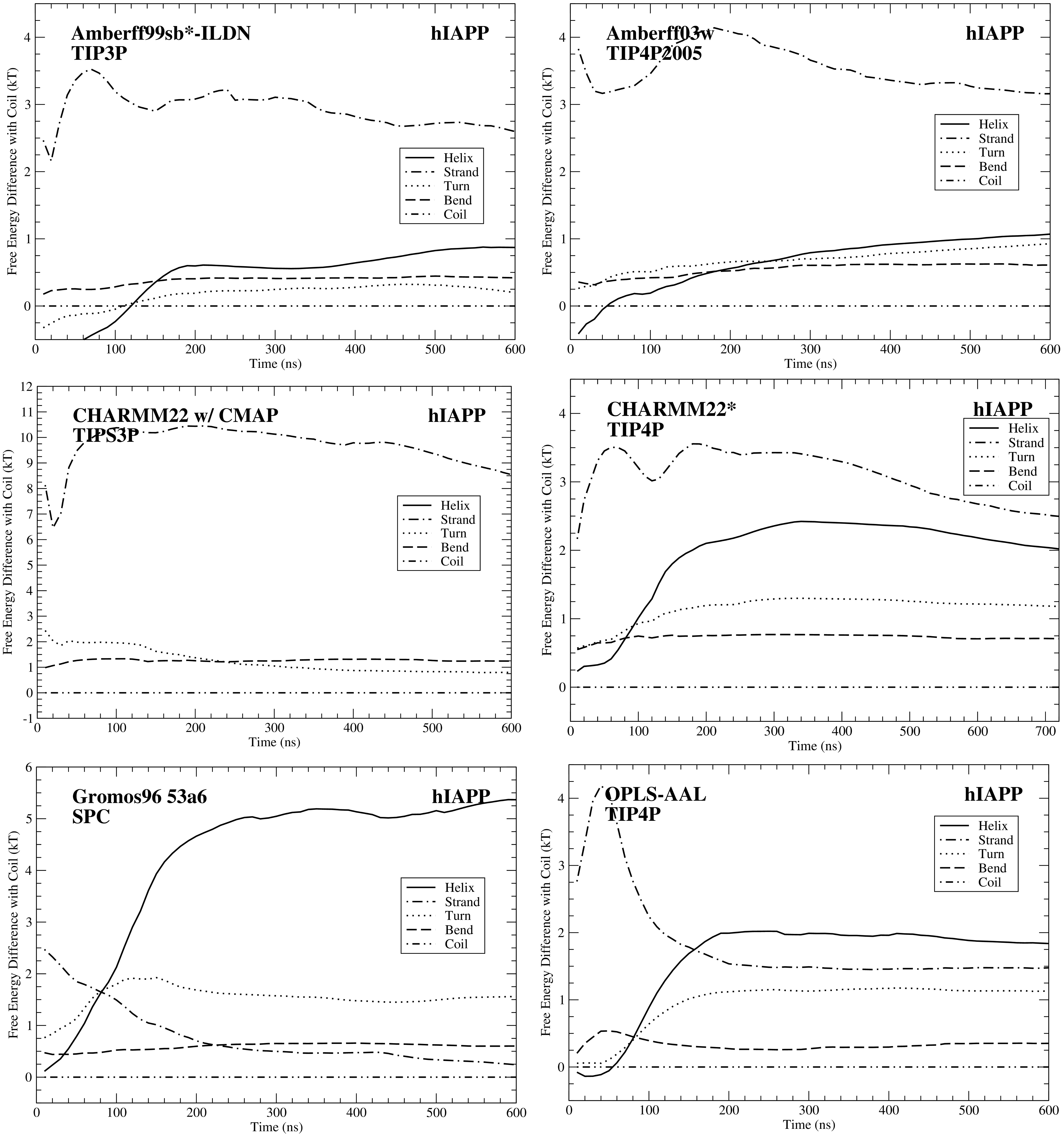

Supplement: S3 Fig — The secondary structure of each residue was determined using DSSP. (TIFF) [file pone.0134091.s004.tiff]
